# Quality Improvement Intervention Using Social Prescribing at Discharge in a University Hospital in France: Quasi-Experimental Study

**DOI:** 10.2196/51728

**Published:** 2024-05-13

**Authors:** Johann Cailhol, Hélène Bihan, Chloé Bourovali-Zade, Annie Boloko, Catherine Duclos

**Affiliations:** 1 Laboratoire Educations et Promotion de la Santé University Sorbonne Paris Nord Bobigny France; 2 Infectious Diseases Unit Groupe Hospitalo-Universitaire Paris Seine Saint Denis Assistance Publique des Hopitaux de Paris Bobigny France; 3 Diabetology Unit Groupe Hospitalo-Universitaire Paris Seine Saint Denis Assistance Publique des Hopitaux de Paris Bobigny France; 4 Laboratoire de recherche en informatique pour la santé University Sorbonne Paris Nord Bobigny France; 5 Public Health Department Groupe Hospitalo-Universitaire Paris Seine Saint Denis Assistance Publique des Hopitaux de Paris Bobigny France

**Keywords:** social prescription, discharge coordination, language barriers, readmission rates, ethnic matching, trust, personalized care, discharge, social determinant, social need, tool, quality of care, readmission, quality improvement

## Abstract

**Background:**

Social prescription is seen as a public health intervention tool with the potential to mitigate social determinants of health. On one side, social prescription is not yet well developed in France, where social workers usually attend to social needs, and historically, there is a deep divide between the health and social sectors. On the other side, discharge coordination is gaining attention in France as a critical tool to improve the quality of care, assessed indirectly using unplanned rehospitalization rates.

**Objective:**

This study aims to combine social prescription and discharge coordination to assess the need for social prescription and its effect on unplanned rehospitalization rates.

**Methods:**

We conducted a quasi-experimental study in two departments of medicine in a French university hospital in a disadvantaged suburb of Paris over 2 years (October 2019-October 2021). A discharge coordinator screened patients for social prescribing needs and provided services on the spot or referred the patient to the appropriate service when needed. The primary outcome was the description of the services delivered by the discharge coordinator and of its process, as well as the characteristics of the patients in terms of social needs. The secondary outcome was the comparison of unplanned rehospitalization rates after data chaining.

**Results:**

A total of 223 patients were included in the intervention arm, with recruitment being disrupted by the COVID-19 pandemic. More than two-thirds of patients (n=154, 69.1%) needed help understanding discharge information. Slightly less than half of the patients (n=98, 43.9%) seen by the discharge coordinator needed social prescribing, encompassing language, housing, health literacy, and financial issues. The social prescribing covered a large range of services, categorized into finding a general practitioner or private sector nurse, including language-matching; referral to a social worker; referral to nongovernmental organization or group activities; support for transportation issues; support for health-related administrative procedures; and support for additional appointments with nonmedical clinicians. All supports were delivered in a highly personalized way. Ethnic data collection was not legally permitted, but for 81% (n=182) of the patients, French was not the mother tongue. After data chaining, rehospitalization rates were compared between 203 patients who received the intervention (n=5, 3.1%) versus 2095 patients who did not (n=51, 2.6%), and there was no statistical difference.

**Conclusions:**

First, our study revealed the breadth of patient’s unmet social needs in our university hospital, which caters to an area where the immigrant population is high. The study also revealed the complexity of the discharge coordinator’s work, who provided highly personalized support and managed to gain trust. Hospital discharge could be used in France as an opportunity in disadvantaged settings. Eventually, indicators other than the rehospitalization rate should be devised to evaluate the effect of social prescribing and discharge coordination.

## Introduction

Worldwide, social inequalities are increasing, as well as the weight of the social and economic determinants on health outcomes [1]. Social prescribing (SP) has been proposed as a public health intervention to tackle social determinants of health in a pragmatic way [2,3]. SP is defined as a link worker providing a set of nonclinical services. First implemented in the United Kingdom in primary health care, SP has expanded to other countries [3-6] and is increasingly experimented with, but not yet in France. In the United Kingdom, SP link workers were integrated into the National Health System in 2019 [7]. Other countries, such as Canada, are also implementing SP in their primary health care system, whose actors call for an increased uptake [8]. SP is seen as a model integrating social and health sectors, and builds on the trust of the “prescription”-based relationship [9]. So far, evaluations of SP’s impact on population health have shown mixed results and have fueled debates on which outcomes to measure [6,10-12]. There seems to be a consensus that evaluations should use both quantitative and qualitative methods, assessing process, experience, and outcomes [6,10,13]. Outcomes considered health and more broadly well-being, and were related to patients and health systems [12]. Since SP’s goal is to favor well-being without focusing on a specific health outcome, the time scale for evaluation might also be much longer than usual [14]. Eventually, a scoping review conducted in Wales revealed a lack of consensus on the terminology used in social prescription practice, leading to miscommunication among professionals, public health decision makers, and the public [15].

In parallel to primary health care, another entry point of SP could be hospital discharge time. Hospital discharge is a transition period of paramount importance, both in terms of hospital efficiency/effectiveness [16] and quality of care [17]. Discharge coordination (DC) has been tested for years, especially in North America and Japan, to reduce the rate of readmission within 30 days, also with mixed results [18-20]. In Europe, concerns over readmission rates are less of a financial concern, but the same lack of coordination issue at discharge remains [21]. Diseases and related treatments are becoming increasingly complex, and multimorbidities represent a challenge in coordination.

This study sought to combine SP and DC in France for the first time and describe its operationalization. A personalized SP/DC intervention was implemented in two departments of medicine at a university hospital. The intervention consisted of coordinating discharge; screening; and, when needed, tackling some of the unmet social needs related to health. We also measured the impact of the combined SP/DC intervention on readmission rates.

## Methods

The SQUIRE (Standards for Quality Improvement Reporting Excellence) guidelines were followed to describe the intervention and to write the paper.

### Context

Avicenne University Hospital, located in an underserved suburb of Paris (Seine-Saint-Denis department), caters to a disadvantaged population comprising the highest proportion of migrants in mainland France. Two departments of medicine were included in this study, one with a chronic disease profile (Diabetology Department [DD]) and one with an acute care profile (Infectious Diseases Department [IDD]). The IDD manages a variety of infections such as community infections (pneumonia, upper urinary tract infections, etc) and immunodepression-related infections. In both units, inpatients had many comorbidities, including a high prevalence of mental health conditions. For transmissible and nontransmissible diseases, there is a well-known link between deprivation and a higher risk of complications, also known as syndemics [22,23]. Rates of readmission within 30 days for inpatients of these two departments as of 2017, the year of project conception, were 5.9% and 1.3% in the IDD and DD units, respectively.

### Intervention

An intervention was defined as a discharge coordinator (DCo) visiting a patient at the time of discharge, delivering a set of nonclinical services tailored to improve patient discharge instruction understanding and uptake (DC), screening the social needs, and setting personalized goals to meet some of the patient’s social needs that could undermine care (SP). Care was defined holistically including its social determinants.

The intervention was delivered in a quasi-experimental way to reduce inclusion bias: the intervention was delivered the same day of the week in each department. Patients who were discharged on other days of the week from the 2 departments or those not interested in the intervention were automatically included in the nonintervention group.

The details of the intervention delivered by the DCo consisted of the following, which were labeled basic DC, DC, SP, or both SP and DC:

- Reviewing diagnosis and postdischarge instructions, ensuring understanding and providing explanations if necessary (basic DC)
- Checking whether the patient had a general practitioner (GP) and, if not, helping him with identifying one (DC)
- Checking whether the patient had financial constraints with following medical prescriptions and, more generally, if there was any social deprivation delivering support services (SP)
- Inquiring how the patient would attend postdischarge hospital visits and prescribed examinations and, if any constraint was identified, helping the patient (SP)
- Checking living conditions (housing, loneliness), substance abuse, mental health, and other perceived needs related to health, and referring patients according to goals set (SP)
- Checking the feasibility of the treatment on a practical level and, if needed, delivering support services (SP/DC)
- Communicating DCOs’ contact information to the patient for them to call the DCo if needed (SP/DC)

If the patient was not fluent in French, the DCo used a translator over the phone and together with the patients identified a trusted person to whom important discharge information would be passed, with the patient’s approval.

The DCo was recruited on a part-time basis for this study. She was a graduated nurse with long experience in patient support in the oncology unit of the same hospital. She was knowledgeable about the context of the hospital patients. She also pursued a postgraduate university diploma in health navigation in 2020. She was under the supervision of 2 medical doctors (one specialized in public health and infectious diseases, and one in endocrinology) for her interventions.

### Data and Population

#### Eligibility to the DCo Intervention

All eligible patients were discharged on Wednesday or Friday in the DD or IDD from October 1, 2019, to June 30, 2021, and on Monday or Thursday for DD or IDD from July 1 to October 30, 2021. The day changed once during the period of intervention due to changes in the units’ organization.

#### DCo Intervention Exclusion Criteria

Patients were excluded if they refused to participate or were already seen before the study.

### Data

A paper-based questionnaire on sociodemographics and literacy items was provided at the beginning of the DCo visit (Multimedia Appendix 1); a paper-based record was kept on the type of support provided by the DCo and on the type and duration of follow-up when needed. Data were then entered into an Excel (Microsoft Corporation) spreadsheet by the DCo.

Data on the SP/DC process was collected through regular follow-up meetings held between the DCo and the 2 medical doctors in charge of the study.

A list of all patients hospitalized in the 2 units during the study period was extracted from the hospital database, with the variable unplanned hospitalization. Only the first hospitalization (nonintervention group) or the hospitalization during which the intervention occurred (intervention group) was kept if several hospitalizations occurred for the same patient. The data set of all patients was chained with the data set of patients who received the SP/DC intervention, using the patient unique identifier number.

### Analysis

The design of our study being quasi-experimental, any difference in outcome is inferable only to the intervention. The sample initially computed for the protocol was 380 patients in the intervention group to be able to show a 5% decrease in readmission rate in the IDD. The readmission rate was calculated for all patients hospitalized in DD or IDD for more than 1 night between October 1, 2019, and October 10, 2021, and who were discharged to home. The outcome was compared between patients in the intervention group versus those who did not receive an intervention. Patients transferred to another facility during or at the end of their stay and patients previously seen by the DCo, in cases of readmission, were not included in the sample.

Characteristics of patients included in the intervention group and type of support services provided were described using descriptive statistics, and chi-square tests were used to run all comparative statistics. The software used was R Studio (2021.09.01; Posit, PBC), and *P* values were considered significant when below the threshold of .05. The process of the interventions, including reflexivity, was analyzed continuously during the study via regular meetings between the 2 supervisors and the DCo and other health professionals involved in the patients’ care when needed.

### Ethics Approval

The Ethical Review Board of the French National Institute for Health and Medical Research (Inserm) reviewed and approved the protocol (IRB00003888, approval 18-534, November 2018).

Patients who were informed about the intervention by the DCo gave their informed consent orally. They were all informed about the possibility of not receiving the intervention and that they could stop the intervention without any consequence on further care. The questionnaire and patient information sheet are presented in Multimedia Appendices 1 and 2, respectively. Data were fully anonymized for the purpose of the study. Only the patient identifier was kept in a file to keep record of those who received the intervention. When the DCo and patient decided on a follow-up intervention, an additional record was created for the DCo to perform the follow-up. There was no compensation provided to participants.

### Patient and Public Involvement

The selecting committee of the funding agency (Agence Régionale de Santé) comprised representatives of the public. At the university hospital level, the project implementation was monitored by a pilot committee constituted of, inter alia, patients’ representatives.

## Results

### Characteristics of Patients in the Intervention Group

A total of 228 patients were eligible for the intervention group, 5 patients refused to be included, amounting to 223 included patients. Reasons for noninclusion consisted of 1 patient stating that he had no need for the intervention and 4 who had to leave since the transportation taking them home had arrived by the time the DCo went to see them.

Socioeconomic and demographic characteristics are presented in Table 1.

Health literacy, access to care, and perceived needs are presented in Table 2.

Regarding language characteristics, the most frequent mother tongues were Arabic (n=56, 25%), French (n=40, 18%), Tamil (n=18, 8%), and Bambara (n=13, 6%). Other mother tongues (n=96, 43%) were scattered between 36 different languages or dialects. When considering patients whose mother language was not French (n=83), the proportion of those not fluent in French increased to 45.6% (n=38). Patients who were not fluent in French originated mostly from Asia (n=27, 33%), sub-Saharan Africa (n=25, 30%), and Northern Africa (n=21, 25%).

**Table 1:** Socioeconomic characteristics (N=223).

		Participants, n (%)
**Education level**		
	None	36 (16.1)
	Primary	39 (17.5)
	Secondary	104 (46.6)
	Tertiary	44 (19.7)
**Income level <€950 (US $1105)**		
	Yes	104 (47.5)
	No	115 (52.5)
**Housing conditions (n=211)**		
	Tenant	111 (52.3)
	Hosted (by family, friends, or other)	51 (24.2)
	Owner	45 (21.2)
	Homeless	5 (2.3)
**One or more children to support (n=201)**		
	Yes	77 (38.3)
	No	124 (61.7)
**Living alone (n=212)**		
	Yes	53 (25.0)
	No	159 (75.0)
**Complementary health coverage (n=189)**		
	None	13 (6.9)
	Mutuelle	136 (72.0)
	Solidarity insurance	40 (21.1)
**Source of income (n=202)**		
	Employment	81 (40.1)
	Retirement pension	55 (27.2)
	Unemployment benefit	32 (15.8)
	Disability allowance	25 (12.4)
	Other	9 (4.5)

**Table 2:** Perceived health-related needs and health literacy characteristics (N=223).

			Participants, n (%)
**Need help to understand medical prescription**			
	Yes	154 (69.1)	
	No	69 (30.9)	
**Need any type of support at discharge to improve health management**			
	Yes	88 (39.4)	
	No	135 (60.6)	
**Fluent in French**			
	Yes	140 (62.8)	
	No	83 (37.2)	
**French is the mother tongue**			
	Yes	41 (18.4)	
	No	182 (81.6)	
**Need help to collect medical prescription at pharmacy (n=207)**			
	Yes	101 (48.2)	
	No	106 (51.9)	
**Perception of financial hardship (n=199)**			
	Yes	87 (43.7)	
	No	112 (56.3)	
**Transportation issues with attending the next medical visit (mobility, financial, etc; n=214)**			
	Yes	17 (7.9)	
	No	197 (92.1)	

### DC and SP Process Analysis

At the beginning of the study, the DCo had to obtain the list of patients who were being discharged from the two departments. The staff responsible for making this list available to the DCo was not fully cooperative, especially in the DD. However, after the 2 supervisors intervened to re-explain the DCo’s role and trust was built between the staff and the DCo, the situation improved.

The DCo managed to establish an empathic relationship with patients at their discharge time. Her presenting as a nurse but acting as a DCo facilitated the relationship. The DCo felt that patients were satisfied meeting her and reviewing the discharge information. The DCo felt that she was always welcome, as if she filled in a vacuum, taking up a role that was missing. She felt useful each time she met a patient.

The DCo shared an ethnic background with a large proportion of patients, which also increased her trustworthiness [24,25]. Her training as a nurse enabled her to discuss with physicians when she faced incomplete medical prescriptions. She liaised with a wide range of actors: social workers and clinicians and administrative staff at the hospital level, social workers of the patients’ hometowns at the community level, nurses providing home visits, patients’ GPs, numerous local nongovernmental organizations (NGOs), and administrative staff. The DCo accompanied each referral to a support service with a cover letter introducing the patient and their needs, and pinpointed at the facility/NGO what was most likely to meet the patient’s needs. She ended her support once the goal was reached, yet patients were free to call her in case another need emerged. The DCo had to adapt her support services over time, depending on the changing span of hospital social worker duties and their availability.

Finally, on the financial side, the intervention costed a total of €35,000 (US $40,698; €31,000 [US $36,046] for a part-time DCo during the 2 years and €4000 [US $4652] for medical supervision time).

### Type of Support Provided by the DCo

Among the 223 patients included in the intervention group, 43.9% (n=98) needed a SP/DC support service, and 125 had only basic DC services. Among the 98 patients, 77 needed a postdischarge follow-up. The median follow-up duration was 8 (IQR 1-25) days. The duration of the DCo intervention at the time of discharge was more than 1 hour for 30% (n=29) of patients and between 30 and 60 minutes for 49.2% (n=48) of patients.

The DCo delivered a total of 119 support services, either on the day of discharge or during the following days. These support services were roughly categorized between DC (n=22), mixed (n=25), and SP (n=72): finding a GP (n=12) or private sector nurse (n=10; DC), referral to a social worker (n=24; SP), referral to NGO or group activities (n=19; SP), support for transportation issue (n=18; DC or SP), support for health-related administrative procedure (n=10; SP), and support for additional appointment with nonmedical clinicians (n=19; SP). The referrals to a social worker included addressing health coverage issues, allowing patients to access medications that were otherwise unaffordable. Each service was personalized, for example, for association referral, looking at the right association near the patient’s home, checking if it was still receiving new beneficiaries and writing a referral letter, applying for complementary health insurance, applying for a health-related residence permit, seeking a GP speaking the same language, etc. Support for administrative procedures meant that the DCo would at times physically accompany the patient to the appropriate facility.

### Comparison Between DCo and Non-DCo Groups

We monitored the implementation of other interventions on SP and DC in the two departments during the study period and identified one specific DC intervention for patients diagnosed with COVID-19 from May 2020. This implied excluding 496 patients with a COVID-19 diagnosis from our study. After excluding them, a total of 2706 patients were extracted for the study period; among those, there were 408 multiple hospitalizations, which were excluded. Among the remaining patients, 203 who received the DCo intervention could be identified via data set chaining, leaving 2095 who did not receive the DCo intervention.

The characteristics of patients in these 2 groups were compared according to their age, gender, residential area, vulnerability score, and duration of hospitalization. No significant differences were found (Table 3 and Multimedia Appendix 1).

Rates of rehospitalization were 3.1% (5/203) in the intervention group and 2.6% (51/2095) in the nonintervention group, with no significant difference (*P*=.99).

The 5 cases of rehospitalization among the patients who received the intervention were reviewed; these cases were related to severe drug side effects (n=2), complications of chronic illness after discharge unrelated to adherence (n=2), and COVID-19 infection (n=1). We also reviewed 5 random patients hospitalized in the nonintervention arm: 3 pertained to complications of chronic illnesses unrelated to adherence, 1 to drug side effects, and 1 initial medical diagnosis error. Hence, the causes of rehospitalization did not differ between the two groups.

**Table 3:** Comparison of characteristics between intervention group (n=203) versus nonintervention (n=2095) group.

		Intervention, n (%)	No intervention, n (%)	*P* value	
**Hospitalization department**					.35
	Endocrinology	135 (66.5)	1465 (70.0)		
	Infectious diseases	68 (33.5)	630 (30.0)		
**Age categories (years)**					.47
	16-24	12 (5.9)	109 (5.2)		
	25-45	48 (23.6)	495 (23.6)		
	46-65	90 (44.3)	839 (40.0)		
	>65	53 (26.2)	652 (31.2)		
**Gender**					.77
	Female	87 (42.9)	870 (41.5)		
	Male	116 (57.1)	1225 (58.5)		
**Social deprivation^a^**					.31
	Yes	133 (65.5)	1450 (69.2)		
	No	70 (34.5)	645 (30.8)		
**Living in Seine-Saint-Denis**					.16
	Yes	180 (88.7)	1776 (84.8)		
	No	23 (11.3)	319 (15.2)		
**Length of hospitalization (days)**					.70
	<10	157 (77.3)	1667 (79.6)		
	11-30	41 (20.2)	374 (17.9)		
	>30	5 (2.4)	54 (2.5)		

^a^If patients declared a specific health assistance coverage.

## Discussion

### Findings Synthesis

Combining SP needs screening and DC at discharge time identified that more than one-third of our sample needed SP. The DCo managed to set goals with patients according to their perceived priorities in a personalized way. Needs were heterogeneous and included wider social needs, which by their nature, impacted the access to and quality of care, with low health literacy levels and a high proportion of language issues. Our study did not show any difference in readmission rates between the DCo and non-DCo groups.

### SP at Discharge: For Whom?

The sample of our study, which is representative of the patients in the two departments, is socially deprived. In France in 2021, 8% of the population lived below the poverty threshold (set at 50% of median income; ie, €950 [US $1105] per month) [26], whereas the proportion in our sample reached 47.5% (n=105). Similarly, the proportion of property owners in France was 58% in 2018 [27], whereas this proportion was only 21.2% (n=45) in our sample. Eventually, the proportion of the French population using the solidarity-based insurance was 10.6% in 2021 [28], and in our sample, this proportion was 21.1% (n=40). The social deprivation of our hospital area population explains the breadth of needs that they face and the proportion needing SP, in addition to the basic DC that everyone received. The high proportion of immigrants (around 30% of the department population) also accounts for the high proportion of patients whose mother tongue was not French. A cost-effectiveness evaluation conducted in a disadvantaged North England area among patients with type 2 diabetes showed that the highest benefit was seen among the most disadvantaged population [29]. This result shows that the SP/DC intervention in our university hospital could be similarly cost-effective given the deprivation level. Moreover, some communities seem to struggle more with understanding French than others, such as those from Asia. Standardized screening tools could be applied at hospital entry to help distinguish patients with complex social needs who require more intensive support from those with only a language issue. Some tools have been validated abroad [30] or in France [31] and could be tested in France.

### DC/SP: By Whom?

Several DC initiatives have flourished worldwide, but their effectiveness tends to be limited or limited to specific categories of patients [32,33]. In France, some public hospitals outsource private companies for DC nurses to facilitate certain types of service delivery around patient discharge [34]. However, those private companies provide only a limited and standardized set of services, and do not provide SP. Additionally, such standardization of DC might not be adaptable to all and especially to some underrepresented populations such as those most deprived who are not eligible for these private companies’ services. SP has not been implemented as such in France and is rolled out under other names like health navigators or health mediators. Their entry point is via a specific disease (HIV, diabetes, etc) [35,36] or via a category of population (lesbian, gay, bisexual, transgender, queer; Romani; etc) [37]. One of the reasons might be the structural divide between the social and health sectors in France. The consequence is a lack of holistic and comprehensive care, with each worker attending only to limited needs. The question of the nature of the worker comes next along with questions about training background, gender, and ethnicity of others and the DCo. In the United States, Kahn et al [38] described diverse missions of nurse case management and reported the complex needs of patients with diabetes and mental illness, as in our study. Is it important that the link worker or the DCo is trained as a clinician? From our study, the DCo used her experience as a nurse at times—more as leverage to talk with physicians and analyze prescriptions. This could be categorized as a medication reconciliation, which is generally provided by a pharmacist. Her knowledge of patients’ context and her professional networks were essential to find the best way to support/empower patients. The DCo missions tended to overlap those of health navigator [39], discharge or care coordinator, and link worker.

### DC/SP: Which Indicators Provide Measurable Impact?

In the published literature, SP has been evaluated using a range of indicators (eg, health related, satisfaction, knowledge, or use of services) [5,12,13,40]. Mixed methods protocols considering process and outcomes, and qualitative and quantitative data, are now increasingly being invoked, making use of the highly personalized and person-centered nature of the practice [6,10]. In our study, readmission rates were not different in the two groups. We could have missed readmissions that occurred in other hospitals, but those would have been equally distributed across the two groups. The absence of difference is due either to low statistical power or to a real absence of difference. Many studies failed as well to show any difference in readmission rates, either for SP or for DC interventions, questioning the relevance of this indicator [41,42]. Moreover, the extremely wide scope of social needs addressed by SP leads to a heterogenous set of outcomes, which adds to the difficulty in structuring the practice of SP. Other indicators such as resilience, well-being [43], and social reconnection were proposed [44].

### Study Limitations

The study started in October 2019 and was disrupted by the COVID-19 pandemic. Subsequent activities were again interrupted by successive pandemic waves. Since patients diagnosed with COVID-19 received specific DC, the total number of patients included in the DCo group was lower than expected. However, the readmission rate decreased due to the decrease in bed availability. Due to the end of funding for the DCo after 2 years, the study ended prematurely. Therefore, our statistical power might be lower than planned. Finally, the two units have different pathways for patients’ care between acute and chronic disease.

Nonetheless, our study is the first study measuring systematic needs for SP in a university hospital in France. The quasi-experimental design provides rigor to our study. Patients receiving the DC intervention might thus be considered as representative of the patients in the two departments, revealing the depth of their unmet needs. The relationship between DCo and the patient was facilitated by the fact that the patient has been hospitalized and gained trust in the hospital, enabling the collection of social needs data in detail and without raising ethical issues since a subsequent support service was provided.

Overall, our study provided a thorough analysis of the process of SP implementation at the time of discharge and contributed to the question of which outcome to measure. Our findings will enrich the current discussion on the structure of social prescription.

### Conclusions

SP is becoming more important in Europe and is considered an innovative tool to switch from a biomedical care to biopsychosocial model of care, with the potential for alleviating social determinants of health. Past evaluations found only low levels of SP effectiveness with regard to health care use. Some studies started to show the complexity of services provided by DCo who also act as social prescribers [45]. Engaging patients in their own DC could also contribute to empowering them [21]. More longitudinal studies are needed to measure other indicators such as well-being, social connectedness, and costs to guide policy makers more effectively. Combining SP in a hospital setting and more generally during health care could be a way to close the gap between the health and social sectors in France. More generally, social prescription at different levels of health care, including in a hospital setting, could be a tool to address social inequalities that are globally on the rise.

## Appendix

Multimedia Appendix 1 Information sheet.

Multimedia Appendix 2 Questionnaire.

Multimedia Appendix 3 Data set.

## Abbreviations

DC: discharge coordination
DCo: discharge coordinator
DD: Diabetology Department
GP: general practitioner
IDD: Infectious Diseases Department
NGO: nongovernmental organization
SP: social prescribing
SQUIRE: Standards for Quality Improvement Reporting Excellence

## Data Availability

The data set is included in Multimedia Appendix 3.
