# Supplementary material for: Quality Improvement Intervention Using Social Prescribing at Discharge in a University Hospital in France: Quasi-Experimental Study
Source: JMIR Form Res. 2024 May 13;8:e51728. doi: 10.2196/51728 (PMC11130777; doi:10.2196/51728)
Supplement: Multimedia Appendix 1 [file formative_v8i1e51728_app1.docx]

Information and consent form

Dear Mrs,

Dear Sir or Madam

The Avicenne Hospital and Paris 13 University invite you to take part in a project on personalized support for your discharge. Participation in the project is entirely voluntary, and your refusal to participate will not affect your care.

The project consists of support for your discharge by a healthcare professional subject to medical confidentiality. She will help you plan and organize and organization of the steps to be taken after discharge, in order to improve continuity of care and coordination. She will ask you questions of a personal nature, which will help us to better understand the typology of Avicenne patients, and to identify any obstacles you may experience in terms of health care. She will give you her contact details afterwards, so that you can contact her at a later stage.

No personal data will be collected. The questionnaire is totally anonymous, and does not include any data that could allow you to be your identification, either directly or by cross-referencing.

This project received a favorable opinion from INSERM's CEEI/IRB (IRB00003888) on 13

November 2018.

The completion of the questionnaire constitutes consent to the research.

Investigor’s details:

Dr Johann Cailhol

Department of Infectious and Tropical Diseases CHU Avicenne

125, route de Stalingrad

93009 Bobigny

Tel: 01 48 95 55 55 (extension 6783)

Éducations et Pratiques de Santé Research Unit, ER 3412, Paris 13

Data protection officer contact details:

Timothée Bonnet, Paris 13 University, CIL

[cil@univ-paris13.fr](mailto:cil@univ-paris13.fr)
