# Supplementary material for: Quality Improvement Intervention Using Social Prescribing at Discharge in a University Hospital in France: Quasi-Experimental Study
Source: JMIR Form Res. 2024 May 13;8:e51728. doi: 10.2196/51728 (PMC11130777; doi:10.2196/51728)
Supplement: Multimedia Appendix 2 [file formative_v8i1e51728_app2.docx]

Discharge coordination and social prescribing questionnaire

Hospitalization setting: diabetology / infectious diseases

Date:

Questionnaire number:

A) Socio-professional aspects

Gender: Male / Female

Age range: <25 [25-45[ [45-65[ >65

Level of general and health literacy

Can write / read French fluently: Yes / No

What is your mother tongue :

Schooling level : None / Primary / Secondary / Tertiary

Do you need someone to help you understand prescriptions or medical information documents from your doctor or pharmacist? Yes / No

2. Social rights

Health coverage: Universal Medical Protection (PUMA) / Medical State Aid (AME) / National Health Insurance (CPAM) or equivalent

Complementary Health Coverage: Complementary Universal coverage (CMU-C) / Solidarity complementary coverage (ACS) / Mutuelle

PASS: yes / no

ALD: yes / no

Do you have a family doctor? Yes / No

If yes, what are his/her contact details:

If no, please explain why:

3. Resources

Financial resources

Employed

Unemployment benefit

Active solidarity income (Revenu solidarité active – RSA)

Retirement pension

Disability pension (AAH)

Other social benefits, please specify:

No income: specify status on the territory or at home, unemployment, homeless... :

Do you receive less than 950 euros per month?

How do you perceive your current financial situation?

You don't have any concern

You have to be careful.

You're having some concerns

You find it very difficult

Housing / accommodation

Individual tenant / Individual owner / homeless

Hosted by: friends family acquaintances, in exchange for money

Living in hostel / Hotel

Other (Please specify):

Family situation

Lives alone: Yes/No

Married/couple / Single

Number of dependent children:

Number of children abroad:

B) PSYCHOLOGICAL/MEDICAL

Are you facing an excessive substance use in your opinion: Yes / No

If yes, please state which substance(s): alcohol / drugs excluding cannabis / tobacco

Over the last 30 days, how many days did you suffer from depression, emotional problems or stress?

Are you regularly followed-up by a psychiatrist or a psychologist? Yes No

What would you like to do to look after your health but are unable to do?

What obstacles are preventing you from doing so?

Concretely, when you leave today:

How and who will pick up your medication?

Will you find it difficult to get to your next consultation at the hospital or with your family doctor?

Will you find it difficult to perform the tests you were prescribed?

May I be of any help with regards to your health and what we discussed?

Total intervention time
